# Cultivation of uncultured marine microorganisms

**DOI:** 10.1007/s42995-021-00093-z

**Published:** 2021-03-15

**Authors:** Fengping Wang, Meng Li, Li Huang, Xiao-Hua Zhang

**Affiliations:** 1 State Key Laboratory of Microbial Metabolism, School of Oceanography, School of Life Sciences and Biotechnology, Shanghai Jiao Tong University, Shanghai, 200240 China; 2 Shenzhen Key Laboratory of Marine Microbiome Engineering, Institute for Advanced Study, Shenzhen University, Shenzhen, 518060 China; 3 Key Laboratory of Microbial Resources, Institute of Microbiology Chinese Academy of Sciences, Beijing, 100101 China; 4 College of Marine Life Sciences and Institute of Evolution and Marine Biodiversity, Ocean University of China, Qingdao, 266003 China

Microorganisms (both pro- and eukaryotes) occur in all marine environments with an estimated abundance of 10^3^–10^10^ cells/cm^3^ in sediments and 10^4^–10^7^ cells/ml in seawater; in these habitats they play key roles in marine biogeochemical cycles (Overmann and Lepleux 2016). Surveys employing molecular marker-based high-throughput sequencing and sophisticated metagenomic approaches have revealed the vast diversity of microorganisms in marine environments and have substantially expanded the tree of life (Castelle and Banfield 2018; Hug et al. 2016; Nayfach et al. 2020). However, many of the novel lineages have no cultured representatives. In fact, most (> 99%) of the marine microorganisms have not been cultured under laboratory conditions (Hofer 2018; Jiao et al. 2020) and are seen as “microbial dark matter” (Lok 2015). Although culture-independent approaches have enabled elucidation of the marine microbial assemblies and their metabolic potentials, pure cultures remain needed for microbiological studies. For example, pure cultures are critical for an understanding of the morphology, ecophysiology, and biochemistry of these organisms, which are key to recognizing their functions in complex environmental processes. Likewise, cultures are needed for discovering new bioactive substances, such as anti-tumor bioactive substances, new antibiotics and other secondary metabolites (Ding et al. 2020).

There are a number of reasons for our inability to cultivate marine microorganisms in the laboratory, including a lack of adequate growth conditions, low growth rates, poor development of colonies, requirements for metabolites generated by other microbes, and the presence of dormant cells (Gutleben et al. 2018; Overmann and Lepleux 2016; Xu et al. 1982). Increasing efforts have been made to culture the uncultured microorganisms. Rapid progress in the development of cultivation techniques has resulted in the successful isolation of a number of novel microorganisms of pivotal importance from marine environments, e.g., the ubiquitous SAR11 marine bacterioplankton clade (*Candidatus* Pelagibacter ubique; Rappé et al. 2002) and an Asgard archaeon at the prokaryote–eukaryote interface (*Candidatus* Prometheoarchaeum syntrophicum; Imachi et al. 2020). Cultivation of a microbe is no longer viewed as an outdated craft in recent years, but a skill that requires not only imagination and perseverance but also the latest technologies. However, establishing a pure culture for an important (e.g., the dominant bacterial and archaeal phylotypes in marine environments) and yet uncultured microbe will still be a major challenge as well as a path to potential benefit. This special issue brings together nine review articles and two research papers that highlight various cultivation strategies for marine bacteria, archaea, and eukaryotic microorganisms. Below, we introduce these papers.

In the ever-challenging marine environment, bacterial taxa may have different abundance and physiological states. In our first paper, based on the bacterial abundance and physiological states, Mu et al. (2021) categorize previously uncultured bacteria into three groups: dominant active bacteria, rare or low-abundance active bacteria, and dormant bacteria. They then propose techniques for isolating each group of uncultured bacteria, i.e., simulating the natural environment for cultivating dominant active bacteria, culturomics and enrichment culture methods for cultivating rare active bacteria, and resuscitation culture for isolating dormant bacteria. By using a modified enrichment culture method (Mu et al. 2018), the authors have isolated and described one novel class (Mu et al. 2020a), three novel orders, five novel families, and over 30 genera of Bacteria. They also found that the isolated bacteria within the order *Bradymonadales* represented a novel group of predatory bacteria that are widely distributed in the saline environments (Mu et al. 2020b).

Microorganisms often occur as complicated multispecies communities in biofilms. Currently, we have limited knowledge of cell-to-cell interactions, within or between species; these interactions may alter the growth of species within the community. In our second paper, to culture biofilm microorganisms, Salam et al. (2021) summarize the progress in cultivation techniques and propose some promising strategies for microbial cultivation in future, including network-directed isolation, culturomics approach, and “Reverse-genomics”. By improving isolation procedures and designing new cultivation techniques (Asem et al. 2018; Qin et al. 2009; Xian et al. 2020), the authors have isolated and described at least 500 novel bacterial and archaeal taxa, consisting of one new class, 23 new orders/sub-orders, 29 new families, and more than 80 new genera.

In situ cultivation technique is one of the most popular techniques for cultivating uncultured microorganisms from marine environments (Overmann and Lepleux 2016). In our third paper, Jung et al. (2021) introduce various in situ cultivation methods, including diffusion chamber, microbial trap, iChip (isolation chip), iTip (in situ cultivation by tip), and double encapsulation techniques. They then propose a hypothesis explaining how in situ cultivation can access previously uncultured microorganisms. By using iChip, the authors cultivated a previously uncultivable marine bacterium that can produce three new diketopiperazines (Ding et al. 2020). In our fourth paper, Liu et al. (2021) further develop a FACS-iChip system by integrating iChip with flow cytometry-based fluorescence-activated cell sorting technique. The results of this study offer a highly efficient and readily available approach to mine uncultivable microorganisms in a nature environment.

Microfluidic droplet-based technique is emerging as a promising new cultivation and screening strategy, with advantages of high-throughput, microscale, single-cell resolution, automation potential, and low cost (Hu et al. 2020; Jiang et al. 2016). In our fifth paper, Hu et al. (2021a) introduce key aspects of droplet microfluidics, including droplet manipulation, operation and detection. They then highlight the applications of microfluidic droplet-based techniques in microbiology, including high-throughput single-cell cultivation of uncultured microorganisms, droplets-based microbial co-cultivation, enzyme screening, as well as single-cell whole-genome sequencing.

The viable but nonculturable (VBNC) state, first described by Xu et al. (1982), refers to a “dormant” state where bacteria are physiologically or metabolically active, but no longer capable of forming colonies on conventional culture media. This is likely a survival strategy to cope with unfavorable environmental stresses, and the bacterial cells in the VBNC state may restore culturability under appropriate conditions (Oliver 2016; Pinto et al. 2015). In our sixth paper, Zhang et al. (2021b) propose that restoration of VBNC cells into culture may be employed as an important strategy to cultivate the not yet-cultured marine microorganisms. They summarize a number of resuscitation stimuli that could be used to culture the previously uncultured marine bacteria, including resuscitation-promoting factors Rpfs and YeaZ, sodium pyruvate, quorum sensing molecules, catalase, and siderophore.

Deep-sea hydrothermal vents (DSHV) host a rich diversity of microorganisms including chemolithoautotrophs, heterotrophs, and/or mixotrophs with versatile metabolic strategies. While large efforts have been made to isolate microbes from this unique environment, limited microbial taxa have been incubated in the laboratory. In our seventh paper, Zeng et al. (2021) review and compile available data and provide a list of isolates from DSHV with their taxonomic and physiological properties. Furthermore, they indicate the potential roles of these microbes in biogeochemical processes. It is noteworthy that the first culture representative of SAR324 was isolated from DSHV and confirmed as a mixotroph capable of sulfur oxidation and alkane oxidation (Wang et al. 2020). The authors further proposed putative novel approaches to bring more DSHV microbes into cultivation, especially by designing new cultivation approaches, real-time monitoring, and utilization of meta-omics data.

Marine sediments host a variety of important, yet uncultivated archaeal groups including the superphylum Asgard, the Bathyarchaeota, the Woesearchaeota, and the ANaerobic MEthanotrophic (ANME) groups within the Euryarchaeota. In our eighth paper, Hu et al. (2021b) report on how they have explored the diversity of such uncultivated archaea using a combination of improved conventional methods, including cell extraction, cell fractionation, enrichment, and roll-bottle isolation. The authors demonstrate these methods by successfully obtaining three co-cultures composed of Bathyarchaeota (subgroup-8) and a bacterial species. This study, therefore, demonstrates that combining and improving conventional cultivation methods can offer a promising means to enrich and isolate slow-growing archaeal groups from marine sediments.

Haloarchaea, affiliated to class *Halobacteria* and phylum Euryarchaeota, are a group of halophilic archaea inhabiting high salt environments and generally requiring over 100–150 g/L NaCl for optimum growth (Oren 2014). While haloarchaea are useful resources for theoretical and applied research, there remain widespread taxa (e.g., Nanohaloarchaea) that have not been isolated in pure culture. In our ninth paper, Cui et al. (2021) summarize the strategies (e.g., using washed agar, detergent-free culture vessels, long incubation times, and reduced nutrient concentration) for isolating halophilic archaea from thalassohaline and athalassohaline environments. They then propose new strategies to cultivate uncultured halophilic archaea.

Mangrove ecosystems are widely distributed in tropical and subtropical coastal areas. They provide many ecological functions, such as protecting coastlines, carbon storage, and maintaining marco- and micro-diversity (Alongi 2014; Zhang et al. 2019). Archaea are becoming an important area of research interest in microbial fields, with many novel archaeal lineages discovered (Baker et al. 2020; Spang et al. 2017). In our tenth paper, Zhang et al. (2021a) summarize the diversity and metabolism of the archaeal community in mangrove ecosystems across the world and find that Bathyarchaeota, Euryarchaeota, Thaumarchaeota, Woesearchaeota, and Lokiarchaeota are the most abundant lineages with potential ecological roles in carbon, nitrogen, and sulfur cycles. In addition, the authors propose some strategies to culture the not yet-cultured archaeal lineages from mangrove ecosystems.

Thraustochytrids are heterotrophic, unicellular marine protists, and have great potentials in accumulating large amounts of bioactive metabolites, including polyunsaturated fatty acids (PUFAs), carotenoids, and squalene. The isolation of novel thraustochytrid strains from marine environments is a major bottleneck for exploring their applications. In our eleventh paper, Lyu et al. (2021) summarize the commonly used methods (e.g., baiting and direct plating methods) for isolation of strains and propose some promising strategies, including dilution plating, co-cultivation, in situ isolation, diffusion chambers, and sponge-explant-based methods, adopted from other microorganisms (i.e., bacteria, archaea, and fungi).

The 11 articles compiled in this special issue offer an extensive, but in no way exhaustive, review on strategies for the cultivation of uncultured marine microorganisms and their application in the isolation of various microbes. We hope that these papers will stimulate further research in this growing field.
